# Comparing Apples and Oranges?: Next Generation Sequencing and Its Impact on Microbiome Analysis

**DOI:** 10.1371/journal.pone.0148028

**Published:** 2016-02-05

**Authors:** Adam G. Clooney, Fiona Fouhy, Roy D. Sleator, Aisling O’ Driscoll, Catherine Stanton, Paul D. Cotter, Marcus J. Claesson

**Affiliations:** 1 School of Microbiology, University College Cork, Cork, Ireland; 2 Teagasc Food Research Centre, Moorepark, Fermoy, Ireland; 3 Department of Biological Sciences Cork Institute of Technology, Cork, Ireland; 4 Department of Computing, Cork Institute of Technology, Cork, Ireland; 5 APC Microbiome Institute, University College Cork, Cork, Ireland; University of Illinois, UNITED STATES

## Abstract

Rapid advancements in sequencing technologies along with falling costs present widespread opportunities for microbiome studies across a vast and diverse array of environments. These impressive technological developments have been accompanied by a considerable growth in the number of methodological variables, including sampling, storage, DNA extraction, primer pairs, sequencing technology, chemistry version, read length, insert size, and analysis pipelines, amongst others. This increase in variability threatens to compromise both the reproducibility and the comparability of studies conducted. Here we perform the first reported study comparing both amplicon and shotgun sequencing for the three leading next-generation sequencing technologies. These were applied to six human stool samples using Illumina HiSeq, MiSeq and Ion PGM shotgun sequencing, as well as amplicon sequencing across two variable 16S rRNA gene regions. Notably, we found that the factor responsible for the greatest variance in microbiota composition was the chosen methodology rather than the natural inter-individual variance, which is commonly one of the most significant drivers in microbiome studies. Amplicon sequencing suffered from this to a large extent, and this issue was particularly apparent when the 16S rRNA V1-V2 region amplicons were sequenced with MiSeq. Somewhat surprisingly, the choice of taxonomic binning software for shotgun sequences proved to be of crucial importance with even greater discriminatory power than sequencing technology and choice of amplicon. Optimal N50 assembly values for the HiSeq was obtained for 10 million reads per sample, whereas the applied MiSeq and PGM sequencing depths proved less sufficient for shotgun sequencing of stool samples. The latter technologies, on the other hand, provide a better basis for functional gene categorisation, possibly due to their longer read lengths. Hence, in addition to highlighting methodological biases, this study demonstrates the risks associated with comparing data generated using different strategies. We also recommend that laboratories with particular interests in certain microbes should optimise their protocols to accurately detect these taxa using different techniques.

## Introduction

The use of Next Generation Sequencing (NGS) for the analysis of complex microbial communities has increased dramatically in recent years. Reasons for this include a continual decrease in cost and an ever greater appreciation of the ability of NGS to more comprehensively characterise microbial communities than traditional culture based methods. NGS has been advantageous in determining the role of the microbiome in disorders like Inflammatory Bowel Disease [1], diabetes [2], and obesity [3], or environmental communities like wetland soils [4] and oceans [5].

There are many methodological choices to be made when conducting a sequence-based microbiome study. These decisions have led to the introduction of a variety of technical variables that affect the compositional signal to various degrees, potentially limiting the ability to investigate the main hypothesis or to compare results relating to communities that are similar but which have been investigated using different methods. Factors such as sampling methods, DNA extraction protocol [6], amplification, purification and quantification [7] along with sequencing depth [8] can significantly impact results. For instance, using different purification and quantification methods can lead to a five-fold difference in sequence counts while a one-step versus two-step PCR method can led to significant differences in alpha and beta diversity between replicates [7].

The majority of microbiome studies have relied on 16S rRNA gene amplicon sequencing. There are nine different variable regions within the prokaryotes ubiquitous 16S rRNA gene (V1-V9), each flanked by highly conserved stretches of DNA suitable for primer binding [9]. Depending on sequencing technology and chemistry it is possible to sequence a number of adjacent variable 16S rRNA gene regions. However, none of the currently available technologies offer full-length gene sequencing at sufficient depth to allow for multiplexing larger numbers of samples on the same run. Unfortunately no standard approach exists for selecting the most appropriate primer pair suitable for all taxa and type of samples, and the decision is often made based on anecdotal evidence and/or advice from the published literature [10], [11], [12].

One of the first considerations before embarking on a microbiota project is to select a sequencing technology. Traditionally, the most common options are Roche 454 GS-FLX, the Illumina MiSeq (lower output, longer reads) and HiSeq (higher output, shorter reads) and the Ion PGM, each offering a series of advantages and disadvantages (see http://www.molecularecologist.com/next-gen-fieldguide-2014/ for a guide). Both the Illumina and Ion instruments utilise a sequencing by synthesis approach where Illumina use DNA templates immobilised on glass slides and optical detection of fluorescently-labelled nucleotides, whereas templates for the Ion Platforms are immobilised in wells on a semi-conductor chip followed by electrical detection of released hydrogen ions. The Illumina and Ion technologies have been compared for amplicon sequencing using various sampling environments, variable regions of the 16S rRNA gene and analysis pipelines. In one case, when stringent quality filtering and lower sequence similarity cut-off when clustering operational taxonomic units (OTUs) were applied on V4 reads sequenced, negligible differences in alpha and beta diversities were observed within and between soil samples when comparing the MiSeq and the PGM [13]. This concordance was further supported when comparing MiSeq and PGM derived microbiota composition as determined by sequencing V1-V2 amplicons generated using a 20-species mock community and human-derived samples [14]. In the latter case it should be noted that, some significant differences were attributed to the PGM failing to produce full-length reads for certain organisms. Furthermore, while not comparing amplicon sequencing and using relatively early versions of sequencing chemistry on an isolated *E*. *coli* species, Loman and colleagues found MiSeq to have lower error rates and longer reads than the PGM, which on the other hand had the fastest turn-around-time [15].

Comparative studies were also conducted to assess the initial potential of the MiSeq to replace the Roche 454 GS-FLX, while also evaluating the effect of the variable region studied. Kozich and co-authors established a dual-index barcoding approach suitable for variable MiSeq read lengths and amplicon regions, in particular V3-V4, V4 and V4-V5 regions [12]. In terms of read quality, MiSeq was either comparable or better than the GS-FLX Titanium, and the V3-V4 better than the V4-V5 region. Another study compared amplicon sequences of seven tandem variable regions produced by the GS-FLX Titanium and Illumina GAII (predecessor of HiSeq) and showed the V3-V4 and V4-V5 primer combinations performed worst and best in terms of classification accuracy, irrespective of the technology used [11]. It is clear that the choice of primers can have a major effect on the outcome, which was also further substantiated by Tremblay and co-authors, as the V6-V8 or V7-V8 regions returned taxonomic composition from a synthetic community that differed to higher degree than what the V4 region did [16].

With the ever increasing number of technological variables that have the potential to have non-trivial effects on microbiota composition analysis, it is critically important to maintain a consistent methodology within studies and when comparing studies, or to have evidence that any inconsistencies that exist do not bias results. A more expensive alternative to 16S rRNA gene amplicon sequencing is shotgun metagenomic sequencing, which bypasses gene-specific amplification and potentially sequences all fragmented DNA, including that from other microorganisms and viruses, in a community. While providing much more information, including encoded functions of the microbiota, the vast amount of sequence data obtained however leads to a new set of challenges in terms of data processing, storage and analysis. For instance, the Illumina HiSeq 2500 platform can yield over 1,000,000,000,000 bp (1 Tbp) of raw sequence data, which may increase several-fold during downstream processing and analysis. Shotgun sequencing is also possible using both the Illumina MiSeq and Ion PGM albeit with less throughput compared to HiSeq. Some non-metagenomic studies have evaluated these platforms and demonstrated comparable results when used to detect blood pathogens [17], diagnose dementia [18], and detect gene variants across four microbial genomes [19].

In the current study we investigated the impact of various amplicon primer combinations and sequencing technologies on the analysis of complex microbial communities. More specifically we compared amplicon and shotgun data generated by Illumina MiSeq, HiSeq and Ion PGM through the use of six human stool samples using two primer sets covering two different 16S rRNA gene regions (V1-V2 [20] and V4-V5 [21]). We also assessed the depth requirements for analysing stool shotgun datasets, and thus if the MiSeq and/or PGM represent suitable alternatives to the HiSeq.

## Materials and Methods

### 16S rRNA gene amplicon sequencing

Stool samples were collected from six elderly individuals and stored at -80°C during the ELDERMET project [22], approved by the Cork Clinical Research Ethics Committee of the Cork Teaching Hospitals (CREC), which granted full approval on the 19th February 2008 (Ref: ECM 3 (a) 01/04/08). Formal written consent was obtained at the time of recruitment, on the basis of an Information Sheet/Safety Statement, following an ethics protocol that was approved by CREC in compliance with pertaining local, national and European ethics legislation and guidelines to best practice. DNA was extracted from stool samples using previously described methods [23], together with a modified Qiagen DNA extraction procedure. Briefly, DNA was extracted using a QIAamp DNA stool Kit with the addition of an initial bead beating step. Microbial DNA from stool samples was used as template for PCR, which contained 25μl Biomix Red (MyBio, Kilkenny, Ireland), 1 μl forward primer (Sigma Aldrich, Dublin, Ireland) (10pmol), 1 μl reverse primer (Sigma Aldrich) (10pmol), template DNA and PCR grade water (MyBio), to a final reaction volume of 50μl. Conditions were optimised so that only 1 band of the correct sizes was obtained and all PCR were completed in triplicate (see S1 Table for primers and further details). Triplicate PCR products were pooled and cleaned using AMPure magnetic bead purification system (1:1.8 DNA:AMPure ratio) (Beckman Coulter, UK). Cleaned samples were quantified using Picogreen Quant-iT quantification and the Nanodrop 3300 (Fisher Scientific, Dublin, Ireland). Samples were subsequently pooled in an equimolar concentration of 10pM and prepared for MiSeq sequencing using standard Illumina protocols. Libraries were mixed with Illumina generated PhiX (20% of 12.5pM) control libraries and were denatured using freshly prepared NaOH and sequenced using a V3 600-cycle kit. For the PGM, libraries were pooled at a concentration of 10pM and sequenced according to Ion PGM protocols.

### Metagenomic shotgun sequencing

For Illumina MiSeq shotgun sequencing, samples were initially tagmented, whereby the Nextera Transposome with sequencing adaptors combines to template DNA resulting in fragmentation of the DNA and the addition of adaptors using the Nextera XT kit from Illumina. A limited 12-cycle PCR was completed during which time sequencing adaptors and indexing primers were added to the DNA. Amplicon samples were then normalized and pooled, followed by sequencing on the MiSeq platform using Illumina protocols for a 2 x 300 cycle run, with an insert size of 400 bases.

Shotgun libraries for Ion PGM were generated according to instructions from the ‘Ion Xpress^™^ Plus gDNA Fragment Library Preparation’ User guide (Publication number MAN0007044). Libraries were sheared, size selected and individually barcoded using the Ion Xpress Barcode Adapters. Following library quantification and equimolar pooling, the Ion OneTouch^™^ 2 system was used to prepare template positive ion sphere particles containing the clonally amplified DNA libraries using the ION PGM^™^ Template OT2 400 Kit, allowing up to 400 bp single-end reads. Enrichment of the template positive ISPs was performed using the Ion OneTouch^™^ ES and an enrichment percentage of 18% was obtained, which was within the range recommended in the ION PGM^™^ Template OT2 400 Kit guide (Publication number MAN0007218). Sequencing was performed on the Ion PGM using an Ion 318v2 chip and the Ion PGM Sequencing 400 kit (guide number MAN0007242).

Shotgun Illumina HiSeq sequencing reads were obtained from the published ELDERMET dataset [22]. The paired-end read lengths were 2 x 90 bp with an insert size of 300 bases. DNA was extracted from samples using the same method as used above.

### Bioinformatic analysis

MiSeq reads were merged and filtered using *join_paired_ends*.*py* in QIIME version 1.8 using the *fastq-join*.*py* tool [24], whereas the single-end PGM reads were not. Demultiplexing of both MiSeq and PGM reads was carried out using *split_libraries*.*py* also on QIIME [21] with default parameters retaining only reads matching the main length distributed (S1 Table) per primer and with an average quality score of Q25 or above. The differences in quality filtering lengths is due to reverse primers being present in the MiSeq reads. Chimeric sequences were removed via USEARCH version 7.0.1090 using the *uchime_ref*.*py* command along with the ChimeraSlayer GOLD database [25]. OTUs were clustered using the QIIME script *pick_closed_reference_otus*.*py* and the RDP database version 11.4. The Mothur implementation of the RDP classifier was used to assign taxonomy from phylum to genus [26] with a bootstrap cut-off of 80%. Any sequences with less than 80% bootstrap values were assigned as unclassified at that particular rank. Species counts for amplicon data were generated using SPINGO with default parameters [27].

All three shotgun datasets reads were aligned to the human genome version 20 (hg20) to filter out human-derived sequences using Bowtie2 version 2.2.3. Illumina HiSeq and MiSeq reads were subsequently quality filtered and trimmed using Trimmomatic version 0.32 [28] and only allowing a quality PHRED cut-off score of at least Q22 across a sliding window of 20 bp. Reads with a minimum length of 30 bp were also removed. Only PGM reads with a quality score of greater than Q15 and longer than 30bp were retained for downstream analysis [29].

All metagenome assemblies were performed using IDBA_UD version 4.1.2 [30] and MetaVelvet version 1.2.02 [31]. Phylogenetic binning was achieved using MetaPhlAn version 2 [32], Kraken version 0.10.5-beta [33] and GOTTCHA version 0.7.5 [34]. MetaPhlAn2 classifies sequences via clade-specific marker genes, Kraken uses exact alignment of *k*-mers and a lowest common ancestor approach, while GOTTCHA maps reads to non-redundant signature databases to classify at multiple taxonomic levels. Genes were predicted using MetaGeneMark version 3.26 [35]. Metaphor was used to predict core and unique genes with thresholds set to 30% amino acid identity across an alignment covering 50% of both sequence lengths [36]. The core and unique genes were then mapped against the EGGNOG database version 4 using BLAST to create functional profiles for each of the samples and datasets retrieving the top hit with an E-value of 1e-5.

### Statistics

All statistical analysis was performed in R version 3.1.3. In each of the heatplots, Spearman correlations, along with Ward D2 clustering, were performed on the relative abundance at genus level of each sample. As the data was largely non-parametric, Spearman correlations were chosen to prevent breaking the statistical assumptions of Pearson correlations. A Mann-whitney test was used to analyse differences in the taxa between clusters. Where necessary, the P-values were corrected for multiple testing using Benjamini and Hochberg [37]. A P-value of <0.05 was considered significant.

## Results

### Microbiota composition

The data generated reflected the different outputs of the three platforms. For the amplicon datasets the PGM produced 57,720 (mean) ± 9,841 (SD) V1-V2, and 33,454 ± 10,488 V4-V5 reads per sample, respectively, while the MiSeq produced 181,758 ± 108,343 V1-V2, and 102,824 ± 22,154 V4-V5 reads per sample, respectively. For the shotgun datasets there was also a marked difference between the three sequencing technologies, with 26,590,475 ± 51,650 HiSeq, 1,352,748 ± 458,483 MiSeq and 962,226 ± 170,251 PGM reads were generated per sample, respectively.

**Fig 1:**
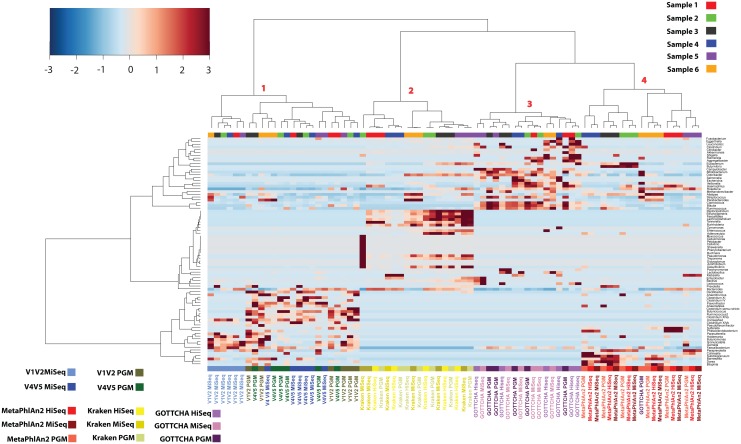
Heat-plot representing the taxonomic composition of the samples at genus level. The heat-plot also includes amplicon data long shotgun datasets from three classifiers namely: MetaPhlAn2, Kraken and GOTTCHA. Only genera in a minimum of 20% of datasets were retained. The method of correlation used was Spearman along with Ward D2 Clustering (PGM = Ion Personal Genome Machine).

We performed hierarchical clustering analysis on the microbiota composition of all six stool samples in order to assess the effect of the amplification primer combination (where relevant), sequencing strategy (16S rRNA gene or shotgun), sequencing technology and type along with metagenomic read classifier. Fig 1 shows a heat-plot with hierarchical clustering of the proportional taxonomic abundances at the genus level, with only genera in a minimum of 20% of the datasets included. All shotgun datasets fell into one large cluster with three distinct sub-clusters, labelled 2, 3 and 4. It is worth highlighting that although the shotgun samples clustered together, there were major discrepancies between the taxonomic profiles (sub-clusters) dictated by the metagenomic classifier used with one exception, sample 6 sequenced on the PGM and classified by GOTTCHA, which clustered with the MetaPhlAn2 sample 6 datasets. In the MetaPhlAn2 cluster (cluster 4), the datasets grouped by sample in each case, which is preferable as it suggests the technical variation is less than the inter-individual variation. For all six samples, the HiSeq and MiSeq datasets clustered together while the PGM sample was located to the side of the sub-cluster. For the GOTTCHA classifier, datasets grouped by sequencer more than by sample. Here there were no case where all three shotgun technologies clustered together by sample. For the third shotgun classifier, Kraken (cluster 2), five of the six samples clustered by sample with the exception of the MiSeq dataset for sample 2. Unlike MetaPhlAn2, the PGM formed sample-wise sub-clusters with HiSeq or MiSeq, with the two Illumina technologies not forming any sub-clusters. Out of a total of 163 genera, 23 were statistically significant between cluster 3 (GOTTCHA) and 4 (MetaPhlAn2) in Fig 1 where the most significant genera included *Ruminococcus* (increased in cluster 3; P-value = 9.88 x 10^−05^), *Blautia* (increased in cluster 3; P-value = 1.30 x 10^−05^) and *Campylobacter* (increased in cluster 3; P-value = 9.30 x 10^−06^). When comparing Kraken, cluster 2, to the other two shotgun classifiers (cluster 3 and 4) there were 52 statistically significant different genera. These included *Buchnera*, *Cellulomonas* and *Cellvibrio*, all increased in the Kraken dataset each with an adjusted P-value of 1.82 x 10^−11^. Of the 15 most significantly different genera, all but one were absent from the GOTTCHA and MetaPhlAn2 clusters, thereby indicating possible false positives detected by Kraken. The three aforementioned taxa are also not predominant colonisers of the human gut thus reinforcing the possibility of inaccuracies in Kraken assignments. See S2 Table for a full list of taxonomy comparisons.

For the amplicon datasets, sample-wise clustering was less prevalent than for the metagenomic datasets. MiSeq V1V2 amplicons were contained in a distinctive sub-cluster, contained within the cluster labelled 1 in Fig 1, clearly separated from the rest of the amplicon datasets. A second sub-cluster contained all the sample 3 and 6 amplicon datasets, with the exception of the V4V5 Miseq dataset and the aforementioned V1-V2 MiSeq dataset. The third sub-cluster contained the majority of the V4V5 MiSeq samples (4 of 6) along with two V4V5 PGM samples. In this case the amplicons clustered by 16S rRNA gene primer combination, as opposed to by sample or by technology. The final sub-cluster contained the majority of the V1V2 PGM datasets (4 of 6) along with 3 of the 4 sample 5 datasets (V1V2 MiSeq being the missing dataset). Investigating the differences between cluster 1 (amplicon data) and clusters 2–4 (shotgun data), uncovered 91 genera to be statistically significant, therefore showing the large differences between amplicon and shotgun classification methods of reads. The full list of taxonomy comparisons are found in S2 Table.

**Fig 2:**
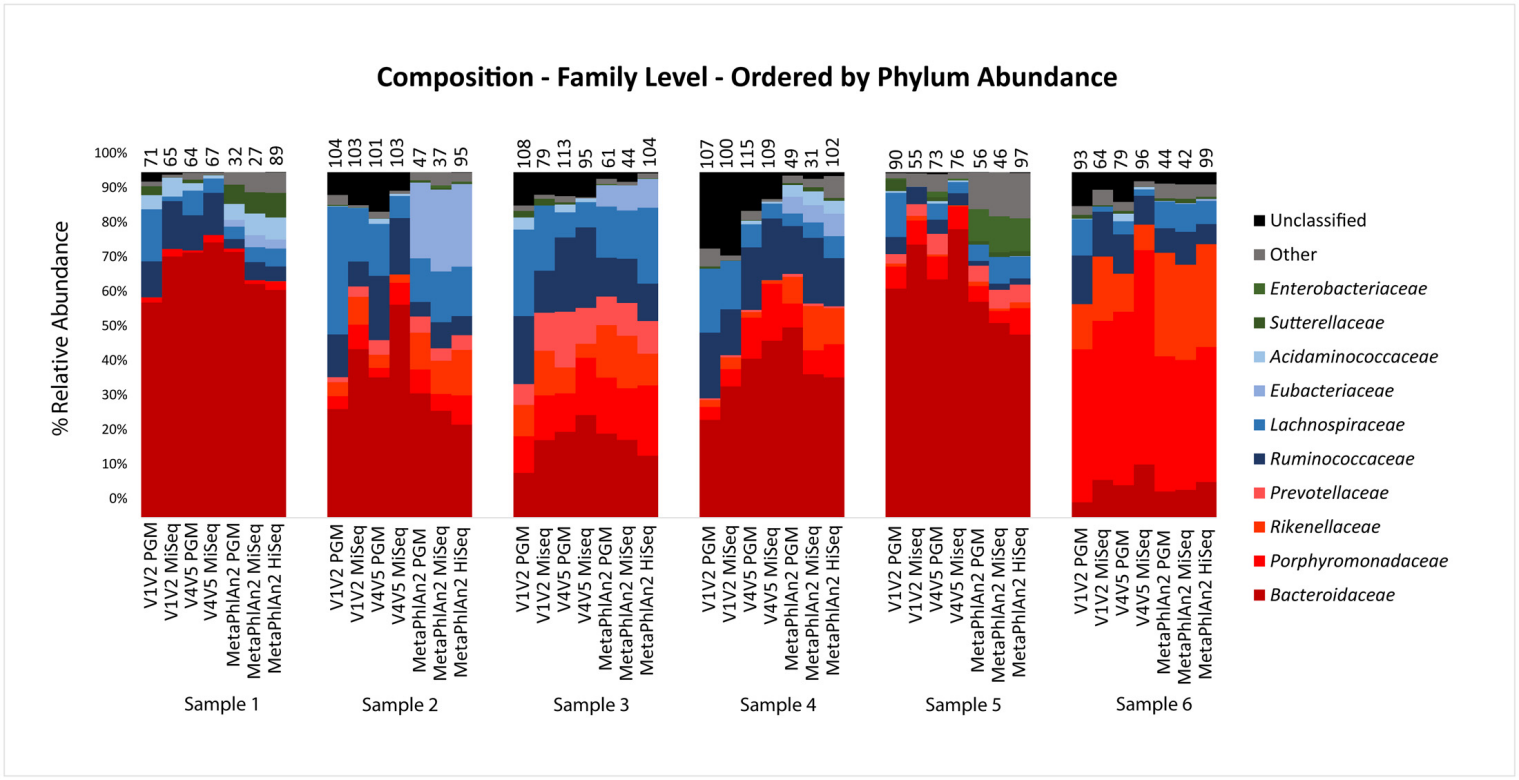
Bar-charts of taxonomic composition at family level. The families are first organised by phylum abundance (highest to lowest) followed by family abundance (highest to lowest) in each of the phyla. The numbers of observed species are located at the top of each bar.

As for bacterial taxa that were the most abundant across all of the datasets, there were some families that differentiate the six subjects regardless of methodology used (Fig 2): For example, *Porphyromonadaceae* genera were consistently high in Sample 6 datasets compared to the other samples, and so were genera belonging to the *Prevotellaceae* family in Sample 3, irrespective of primer combination or sequencing technology. For samples 1 and 5 the shotgun-based methods appeared more sensitive with respect to detecting *Enterobacteriaceae* genera within the *Proteobacteria* phylum compared to the amplicon-based approaches, which could be attributed to the difficulty of discriminating such taxa at 16S rRNA gene level.

**Fig 3:**
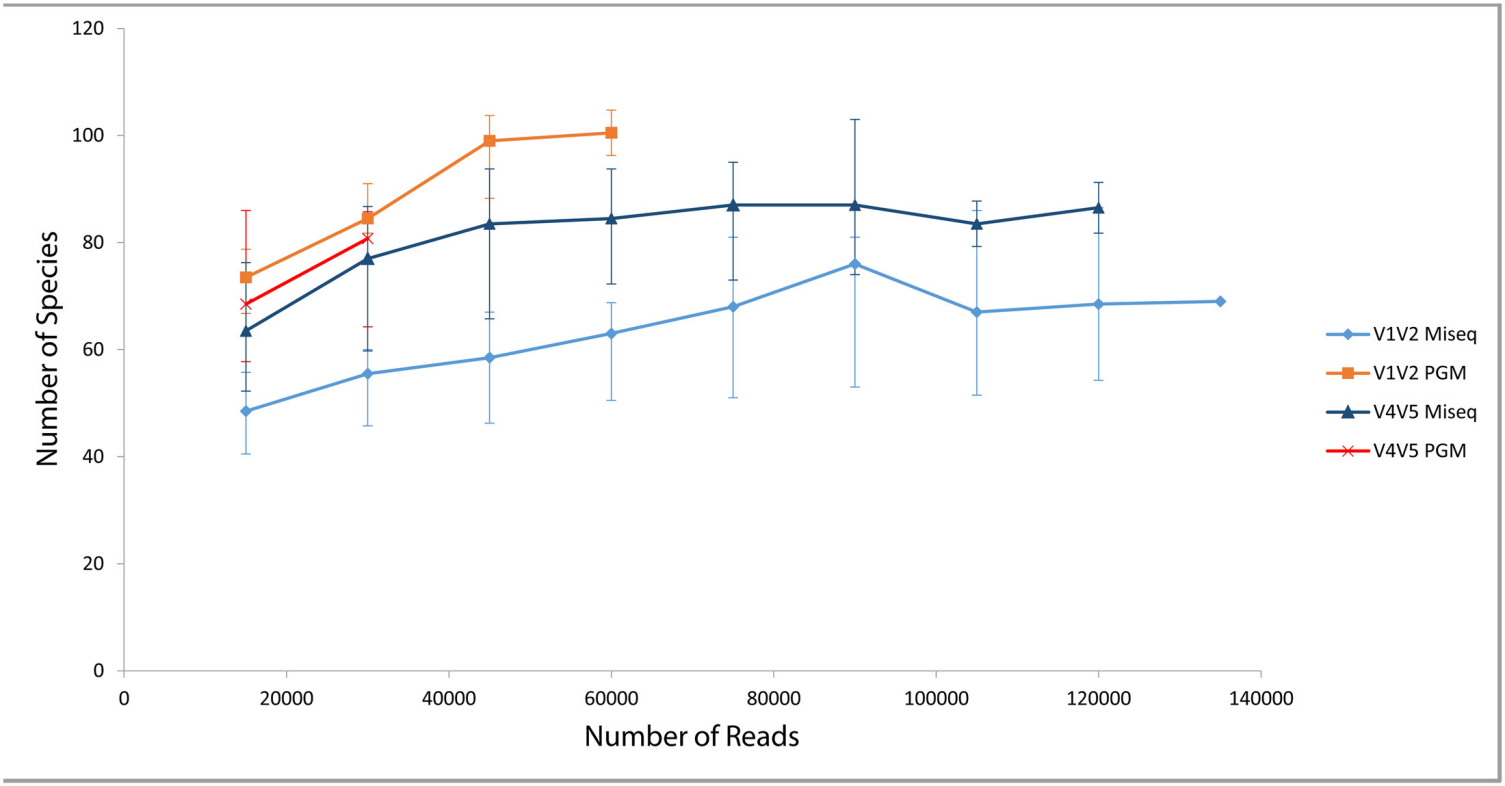
Observed Species at various sequencing depths for the amplicon data using SPINGO. The data points represent the median values across the 6 samples and the error bars are the 25% and 75% quartile ranges.

Fig 2 also highlights the number of unique species in each dataset, as identified by MetaPhlAn2 for shotgun data and SPINGO for amplicon data. Note that these were species that could be confidently classified as such, and should not be mistaken as number of unique OTUs. The highest numbers of unique species among all shotgun methods were detected in the HiSeq datasets, comparable to those resulting from the analysis of amplicons. The success of the HiSeq with respect to shotgun sequencing is not surprising given the greater sequencing depth it can provide resulting in detection of rarer species. The lowest number of unique species overall was detected in the MiSeq shotgun datasets, which is not due to total number of reads as PGM had fewer of these. For the amplicon datasets, the highest number of unique species was detected with the PGM datasets for five of the six datasets. Although the species counts for the pooled PGM amplicons was higher when compared to the MiSeq amplicons, the difference was not statistically significant (P-value = 0.24). However, when comparing particular primer combinations, the difference in the V1-V2 species counts between the two technologies was significant at the 10% level (P-value = 0.093). We further analysed the effect of varying sequencing depth on the number of unique species detected for each amplicon run (Fig 3). The highest numbers of species were detected at each read depth by the V1V2 amplicon on the PGM, while the lowest was the V1V2 on the Illumina MiSeq. All primer datasets reached saturation in the number of new species detected, other than the V4V5 primer on the PGM which was limited by the number of reads for some samples. However, despite this, more unique species were detected with this primer/technology combination than both MiSeq datasets, which had vastly more reads.

### Shotgun sequencing depth

**Fig 4:**
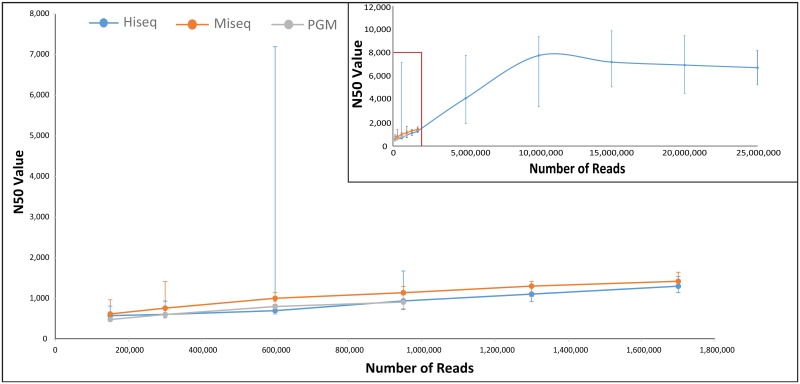
N50 values representing randomly subsampled reads at various sequencing depths after assembly by IDBA_UD. Each point represents the median value across each of the 6 samples per technology (including 3 replicates per sample). Error bars are the 25% and 75% quartile ranges.

To investigate which technology was most suitable for shotgun sequencing, we performed random subsampling of reads to determine occurrences at even sequencing depths, in recognition of the fact that the HiSeq coverage was substantially higher than the coverage for MiSeq and PGM. Fig 4 shows the median N50 values across each of the six samples per technology, including three replicates (random sub-samplings) for each sample. At the lowest sequencing depth selected (150,000 reads) the assembly using the MiSeq data had the highest N50 (minimum contig length above which 50% of all reads are assembled into), possibly due the longer read lengths. However, as more reads were added, the HiSeq data began to outperform the assembly from both the MiSeq and the PGM technologies. The MiSeq and PGM datasets became limited by read number and their N50 value plateaued at 1.7 million and 950,000 reads, respectively. Due to the large number of HiSeq reads, the N50 peaked at 10 million reads after a large increase at 1.7 million reads. Two of the six HiSeq datasets (Samples 1 and 5) had a very large N50 at 600,000 reads. In order to ensure that the results were not affected by the assembler selected, the datasets were also assembled using both Velvet (S1 Fig) and MetaVelvet (S2 Fig). Interestingly, the same two samples for the HiSeq datasets had an elevated N50 for both Velvet and MetaVelvet, however at 1.3 million and 950,000 reads respectively (S3 Table).

**Fig 5:**
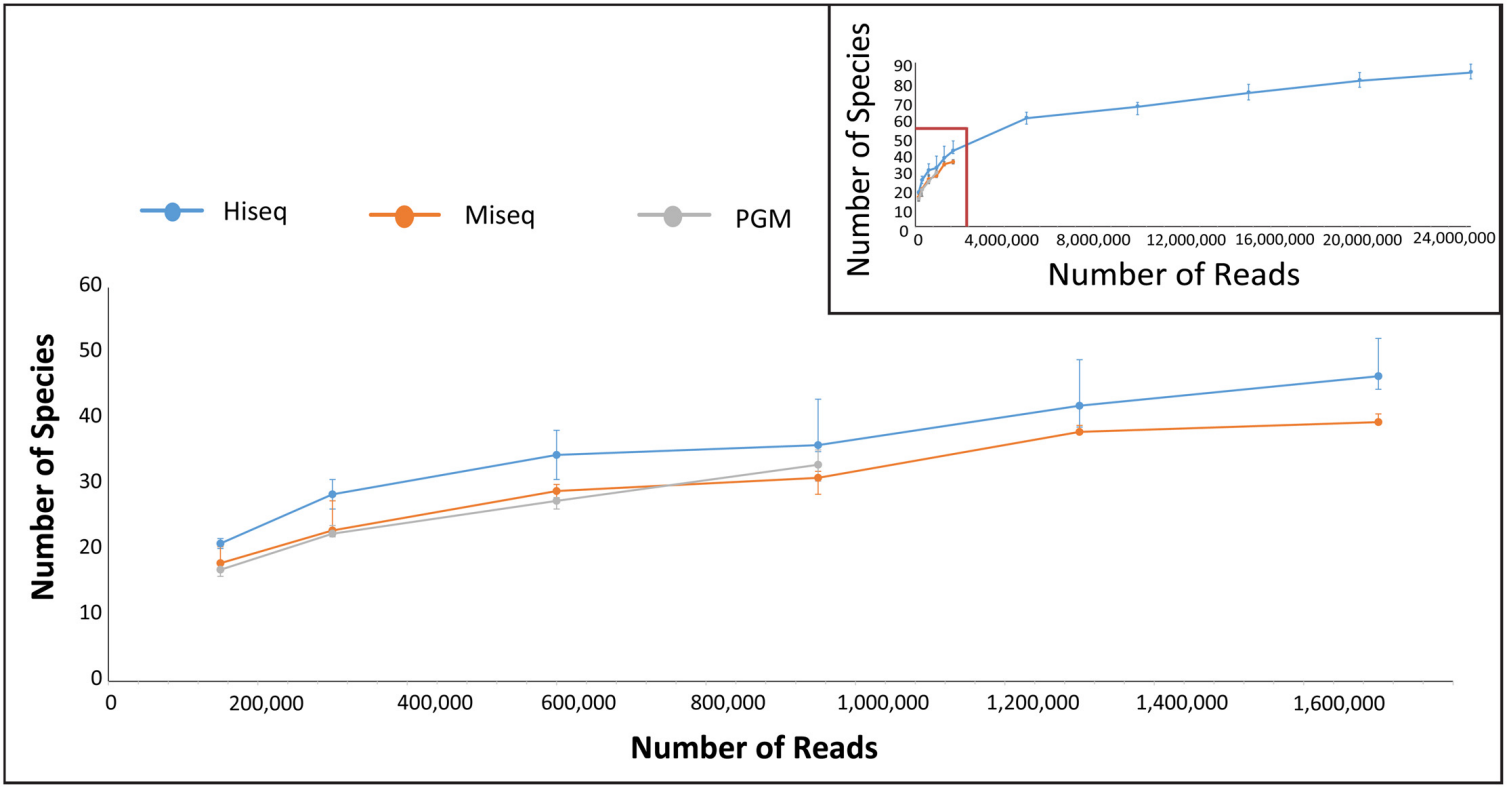
Number of species observed from randomly subsampled reads using MetaPhlAn2. Each point represents the median value across each of the 6 samples per technology (including 3 replicates per sample). Error bars are the 25% and 75% quartile ranges.

Furthermore, unique species detection was also performed on the sub-sampled shotgun sequencing-derived reads (Fig 5). At low sample depths the HiSeq, MiSeq and PGM datasets were comparable with few differences in the number of species detected. At 950,000 reads, the PGM data reached the read limit, but was still similar to the other technologies in terms of number of species. However, at 1.7 million reads, the HiSeq species counts continued to increase while the MiSeq counts level off. This could possibly be due to the fact that the longer MiSeq read lengths result in more accurate species assignments relative to HiSeq, leading to earlier plateauing. In the overall graph (Fig 5 insert) the HiSeq counts continued to increase without levelling off completely even at the 25 million read point.

### Encoded functions

**Fig 6:**
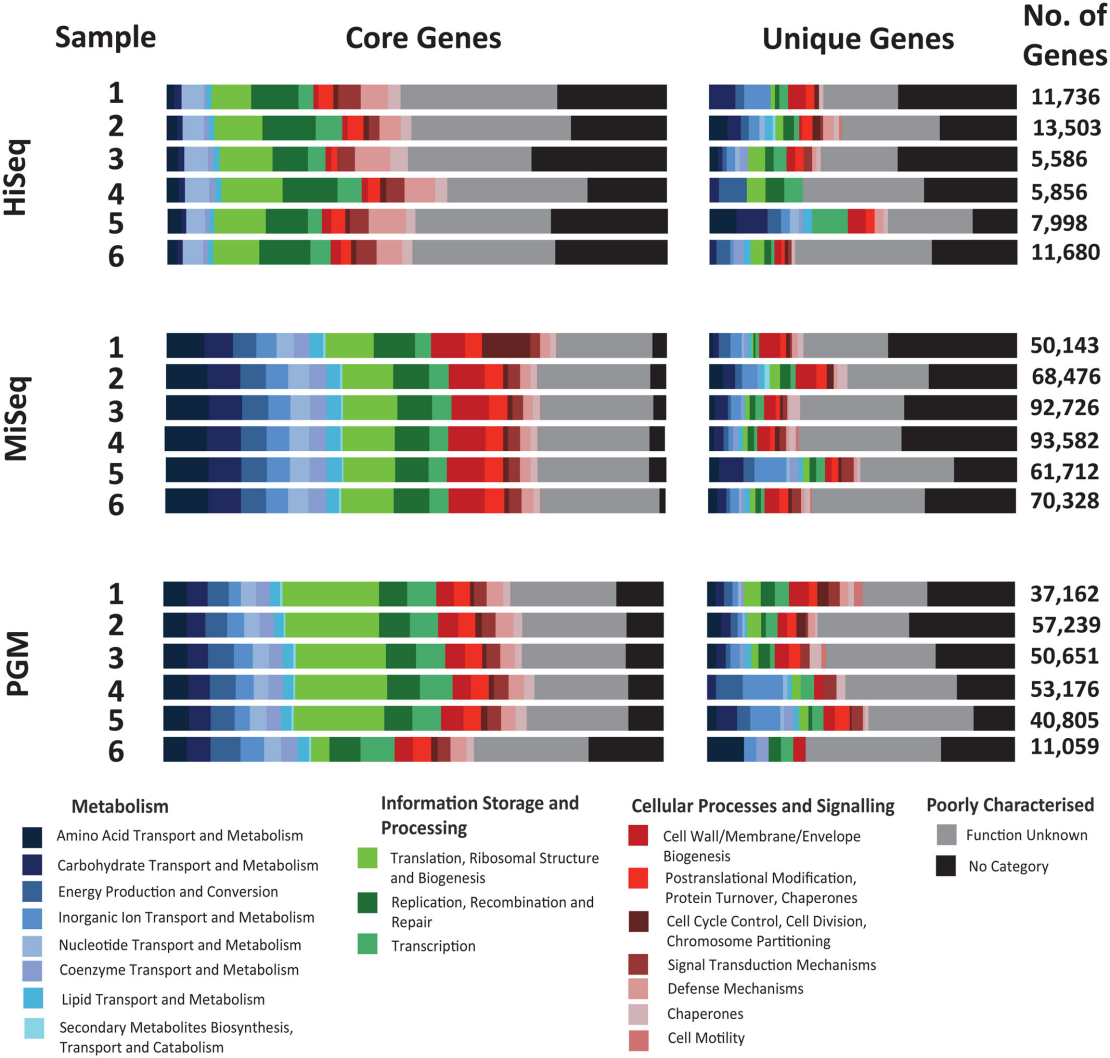
Core and unique genes acquired by Metaphor with 600,000 sequencing randomly selected datasets for each of the samples. The numbers represent the total number of predicted complete or incomplete genes for each metagenome.

From within the categories of shotgun datasets, the core and unique genes were predicted using Metaphor (Fig 6). This was carried out on 600,000 reads per dataset in order to allow for comparative results at equal sequencing depth. For the core genes all three technologies gave broadly the same results, however the HiSeq data had the most poorly characterised genes out of the three datasets, along with the lowest number of genes with a “Metabolism” function and the highest with no function. Surprisingly, this technology did not predict any core genes for the categories, “Energy Production and Conversion” or “Inorganic Transport and Metabolism”, whereas both of these categories were present in the core gene profiles of the MiSeq and PGM datasets. The MiSeq datasets predicted the highest number of genes within the “Metabolism” category, while the PGM data predicted the highest for “Information Storage and Processing”, whilst also being the only technology to predict core genes in the category “Cell Motility”. The number of genes predicted by MetaGeneMark are listed in Fig 6. At a read depth of 600,000 sequences, the MiSeq datasets predicted the most genes for each of the 6 samples while the HiSeq datasets gave the lowest gene number of 5 of the 6 samples. This is a possible reason why this technology gives the most detailed core and unique gene profile.

## Discussion

The NGS technologies Illumina MiSeq, HiSeq and Ion PGM have shown significant promise in delivering cost-effective, high-resolution insights into microbiomes from various environments. However, due to a multitude of technical variables, careful comparisons are required to provide recommendations for suitable methodological approaches. In response to this, we compared the taxonomic composition of six stool samples using two different primer combinations covering two 16S rRNA gene variable regions. We then compared these results with those of shotgun sequencing using Illumina and Ion technologies.

Following either OTU clustering of amplicon reads or taxonomic classification by binning of shotgun reads, all at genus level, we compared microbiota composition of the different datasets. Even though the gut microbiota is generally regarded as individual specific, it was apparent that some amplicon datasets clustered according to technology and/or primer set, rather than by subject. In particular, microbiota composition from all V1V2 MiSeq and four of the six V4V5 MiSeq datasets grouped together in separate sub-clusters. The V1V2 and V4V5 PGM datasets clustered by sample opposed to technology in 3 of the 6 samples (samples 1, 3 and 6) while the V4V5 MiSeq data clustered with V4V5 PGM data per sample in 2 of the 6 samples (samples 5 and 6).

To ensure that the differences in classifications between shotgun and amplicon sequencing were not simply due to a particular shotgun classification method, we compared the compositional clustering with three classifiers of shotgun reads, MetaPhlAn2, GOTTCHA and Kraken. The shotgun datasets grouped together in a sub-cluster separated from the amplicon datasets, which might be expected as these methods are independent of amplification bias and 16S rRNA gene copy number differences. With MetaPhlAn2, all Illumina HiSeq and MiSeq datasets were consistently closer to each other than to the PGM shotgun sequences. This is seen to a smaller degree with GOTTCHA, where three of the six samples sub-clustered the Illumina technologies, but not at all for Kraken assemblies. In terms of clustering by sample over method, MetaPhlAn2 gave the most optimal results with all datasets clustered by sample groups, closely followed by Kraken where this occurred for 5 of the 6 samples in separate sub-clusters. GOTTCHA failed to cluster any dataset by samples, indicating its higher sensitivity for technological artefacts between sequencing methods. However, it must be noted that measuring accuracy based on individual sample clustering is not always a reflection of performance, as GOTTCHA datasets clustered more closely to MetaPhlAn2 and although sample clustering is observed when using Kraken, many of the taxonomic assignments may be false positives as previously mentioned.

Unsurprisingly, Illumina HiSeq shotgun sequences translated to the highest number of species, compared to the other two shotgun datasets, which were more than an order of magnitude smaller. Sub-sampling that simulated lower HiSeq coverage revealed, however, that even equal number of reads could result in more observed species for HiSeq. As this technology produces shorter reads compared to MiSeq and PGM it is possible that the number of species is artificially inflated as a result of higher sequence variation created from incorrect alignment to the reference marker genes. While not directly comparable with species observed through shotgun sequencing, V1-V2 amplicons, which are expected to be more variable than V4-V5 amplicons, sequenced by PGM resulted in the highest species counts.

Despite having the largest number of reads per sample, the V1-V2 region on the MiSeq had at each subsampling point the lowest number of unique species identified. This could be due to the questionable reliability for this primer combination in relation to unexpected clustering and failure to detect expected genera. Curiously, Salipante *et al*. [14], found that sequencing using the same V1-V2 primers on the PGM led to higher error rates when compared to the MiSeq, particularly for a mock community of 20 organisms where deviating abundances of single strains have much greater effect on the overall community composition than in a high-diversity sample. Other reasons for the different results in Salipante et al. study could be attributed to discrepancies in amplification (one-step PCR reaction) and taxonomic assignment (older RDP-classifier version and BLAST).

The benefits to using metagenomic shotgun over amplicon sequencing are clear in terms of increased information content and reduced biases related to amplification and gene copy numbers. However, it is currently not established what sequencing depth is required for the different technologies; this is a more pertinent issue for shotgun than for amplicon sequencing, due to its much higher cost per sample. We therefore assembled the randomly sub-sampled shotgun datasets and compared the common N50 metric across the three sequencing technologies. As expected, the MiSeq technology, with its non-overlapping 300 bp paired-end reads, had marginally higher N50 values than HiSeq and PGM. An N50 peak occurred at 10 million reads for the HiSeq data suggesting that this was the optimal point for sequencing depth for stool samples and 100 bp paired-end reads with 300 bp insert size. There was no peak observed for the PGM or the MiSeq in the available coverage range, which may suggest that the coverage may not be sufficient to reach an optimal level of assembly. Somewhat surprisingly, for two of the six samples there were drastically elevated N50 values at 600,000 HiSeq reads, irrespective of which random sub-sampling set. Such early N50 peaks were also observed using two other assemblers, albeit for a different number of reads, and has previously been reported when assembling sub-samples of an isolated bacterium [38]. In that case, the authors reasoned that this could be due to chimeric reads, duplications or sequencing errors, and recommended that assembled contigs should be incrementally assembled in sub-sections before a final merge. We also suggest that for our data, this read depth may be where the majority of high abundant species are assembled and as more rare taxa are added the assembly becomes less efficient.

In terms of functional categorisation of assembled shotgun sequences, we found the MiSeq and PGM datasets to largely contain equal proportions of predicted core genes from the assembled contigs. For the HiSeq assemblies there were, however, substantially fewer core genes involved in “Metabolism” and more genes with unknown function. This may be attributable to the fewer number of predicted complete genes, which is plausible for this shorter-read technology.

To summarise, this is, to our knowledge, the first reported study comparing both amplicon and shotgun sequencing for Illumina and Ion technologies. Although shotgun sequencing did not suffer from the same degree of technology-dependent bias seen with the amplicon sequencing, there were some major distinct differences between phylogenetic binning software, with MetaPhlAn2 producing the most favourable results. GOTTCHA failed to cluster any datasets by sample, however sub-clustered with MetaPhlAn2, while Kraken clustered separately from the other two binners and also appeared to produce a high number of false positive taxonomic assignments. The variation of microbiota composition between the majority of gut samples proved to be lesser than between the compared sequencing technologies and variable 16S rRNA gene regions. In particular, the V1-V2 MiSeq showed poor performance, while the V4-V5 region was marginally more reliable on both platforms. There is evidence that the MiSeq and PGM offer valuable information when used for shotgun sequencing, however, in order to detect the majority of species in samples and to perform a high quality assembly, deeper sequencing is required. Species assignment is also dependent on read length, which is shorter for the HiSeq. We subsequently showed that there may be no assembly-related benefit in sequencing greater than 10 million HiSeq reads per stool sample. Nevertheless, as the cost of shotgun sequencing is lower on the HiSeq instrument compared to MiSeq or PGM, this platform may still be preferable even though MiSeq produces longer reads and somewhat better assemblies at low sequencing depth. Caution should however be applied with regards to taxonomic binning, and comparisons such as those described in this study must be carried out to prevent methodological biases eclipsing the true biological picture. Hence, we advise laboratories with particular interests in certain microbes to optimise their protocols to accurately detect these taxa using different techniques.

## Supporting Information

S1 Fig N50 values representing randomly subsampled reads at various sequencing depths after assembly by Velvet. Each point represents the median value across each of the 6 samples per technology (including 3 replicates per sample). Error bars are the 25% and 75% quartile ranges.
(PDF)

S2 Fig N50 values representing randomly subsampled reads at various sequencing depths after assembly by MetaVelvet. Each point represents the median value across each of the 6 samples per technology (including 3 replicates per sample). Error bars are the 25% and 75% quartile ranges.
(PDF)

S1 Table PCR primer, linker and adaptor sequences used for sequencing samples on the PGM Ion Torrent and Illumina MiSeq. The table also contains the PCR conditions for 16S rRNA gene amlification and sequence length for quality filtering during read processing.
(XLSX)

S2 Table Statistical comparisons of taxonomic assignments between the various clusters from Fig 1. A Mann-whitney test was used to analyse differences and the P-values were corrected for multiple testing using Benjamini and Hochberg.
(XLSX)

S3 Table The N50 values obtained post assembly, via IDBA_UD, for the PGM Ion Torrent, Illumina HiSeq and MiSeq at various read sub-sampling depths. (XLSX)
