# Supplementary figures and images for: Comparing Apples and Oranges?: Next Generation Sequencing and Its Impact on Microbiome Analysis
Source: PLoS One. 2016 Feb 5;11(2):e0148028. doi: 10.1371/journal.pone.0148028 (PMC4746063; doi:10.1371/journal.pone.0148028)

● Hiseq    ● Miseq    ● PGM

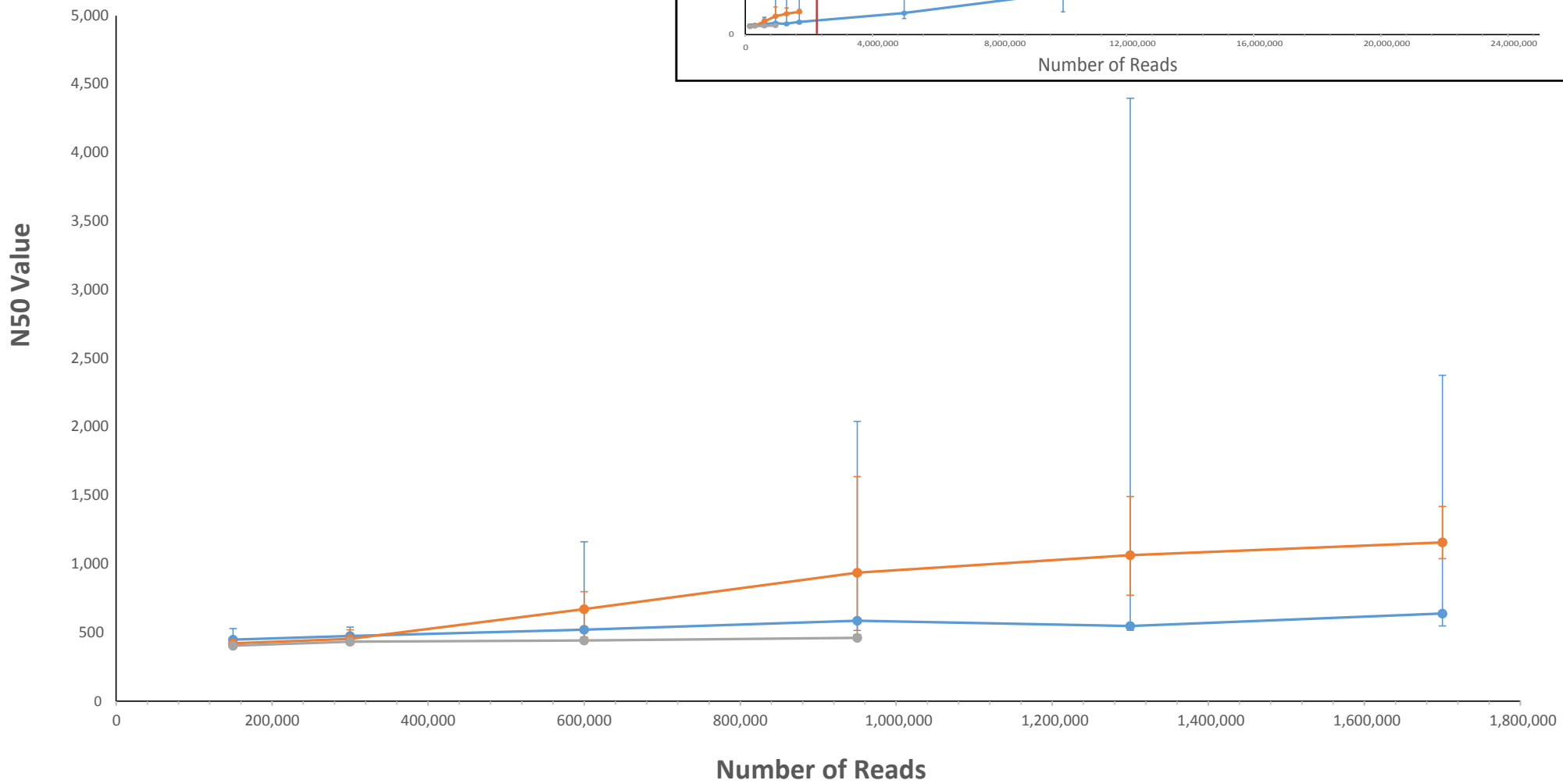

Supplement: S1 Fig — Each point represents the median value across each of the 6 samples per technology (including 3 replicates per sample). Error bars are the 25% and 75% quartile ranges. (PDF) [file pone.0148028.s001.pdf]

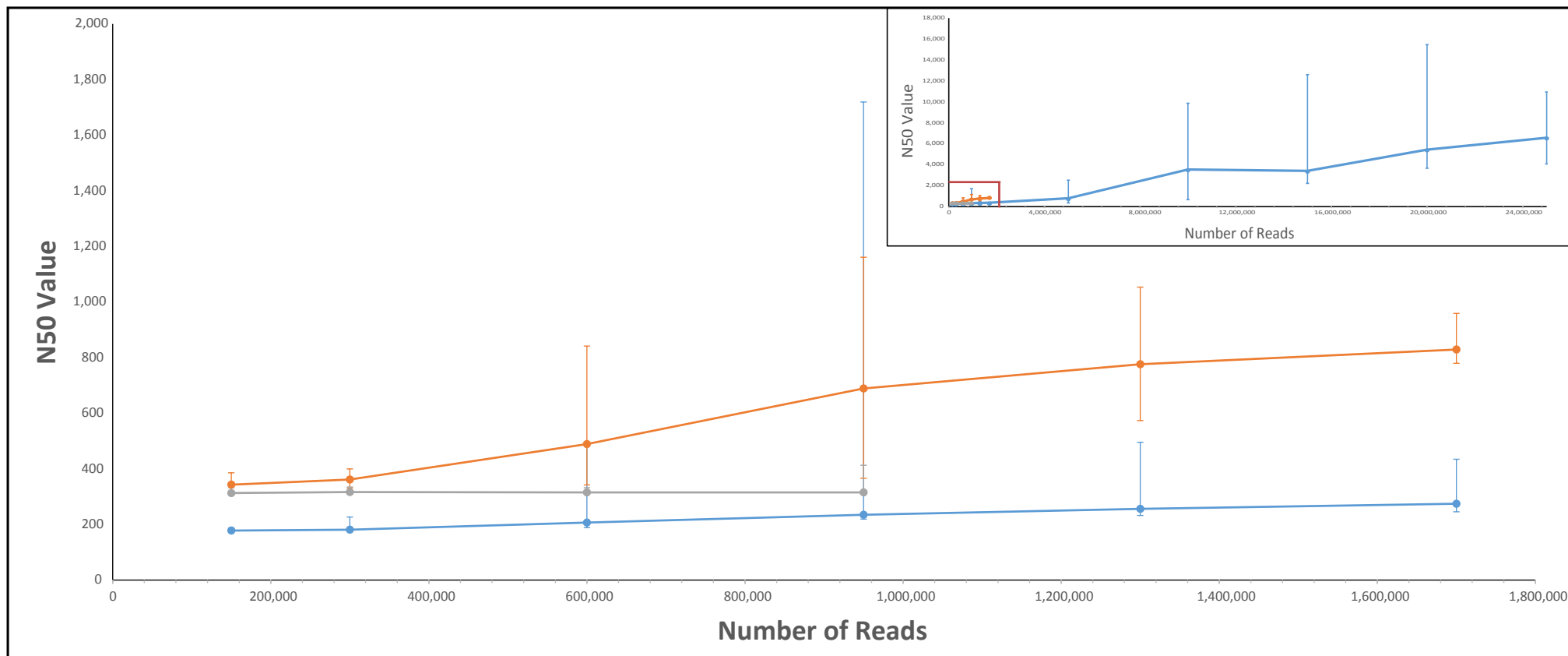

Supplement: S2 Fig — Each point represents the median value across each of the 6 samples per technology (including 3 replicates per sample). Error bars are the 25% and 75% quartile ranges. (PDF) [file pone.0148028.s002.pdf]
